# Clinical Efficacy of Huangkui Capsule Plus Methylprednisolone for Immunoglobulin A Nephropathy and Its Effect on Renal Function and Serum Inflammatory Factors

**DOI:** 10.1155/2023/3020033

**Published:** 2023-02-21

**Authors:** Lili Yuan, Kan Cai, Yueji Zou

**Affiliations:** ^1^Department of Nephrology, Yantai Penglai Traditional Chinese Medical Hospital, Yantai, China; ^2^Department of Radiology, Yantai Penglai Traditional Chinese Medical Hospital, Yantai, China

## Abstract

**Objective:**

To assess the clinical efficacy of Huangkui capsule plus methylprednisolone for immunoglobulin A (IgA) nephropathy and its effect on renal function and serum inflammatory factors.

**Methods:**

A total of 80 patients with IgA nephropathy admitted to our hospital from April 2019 to December 2021 were recruited and assigned (1 : 1) to receive either conventional drugs + methylprednisolone tablets (observation group) or conventional drugs + methylprednisolone tablets + Huangkui capsules (experimental group), with 40 patients in each group. Outcome measures included clinical efficacy, renal function indices, serum inflammatory factor levels, and adverse events.

**Results:**

The experimental group showed a significantly higher clinical efficacy versus the observation group (*P* < 0.05). Patients in the experimental group had significantly lower serum creatinine, serum urea nitrogen, fibrinogen, and 24 h urine protein levels than those in the observation group after treatment (*P* < 0.05). After treatment, the experimental group showed lower levels of tumor necrosis factor-*α* (TNF-*α*), interleukin-6 (IL-6), and monocyte chemoattractant protein-1 (MCP-1) than the observation group (*P* < 0.05). The differences in the adverse events between the two groups did not come up to the statistical standard (*P* > 0.05).

**Conclusion:**

Huangkui capsule + methylprednisolone provides a feasible therapeutic option for IgA nephropathy by considerably boosting patients' renal function, successfully lowering the inflammatory response, and producing a good safety profile.

## 1. Introduction

Immunoglobulin A (IgA) nephropathy is a common clinical glomerulonephritis characterized by the deposition of immune complexes in the glomerular thylakoid region [1]. The main feature of the disease is the deposition of IgA-dominated immune complexes in the mesangial area of the glomerulus, which is shown by renal immunopathology. The clinical manifestations are various, with hematuria as the major one, accompanied by different degrees of proteinuria, kidney function damage, etc. It can even progress to end-stage renal disease, posing a serious threat to the health of patients. The clinical manifestations of the disease are acute and chronic nephritis syndrome, nephrotic syndrome, and hypertension. The current clinical treatment mainly includes nonimmune symptomatic supportive therapy and immunosuppressive therapy [2].

Methylprednisolone is an adrenal glucocorticoid that is effective in improving immune function in patients with IgA nephropathy [3]. Reduced renal function in patients with IgA nephropathy triggers a deterioration of all functional systems of the body and further worsens the condition of patients [4], and Patel et al. [5] indicated that the effectiveness of conventional drugs plus methylprednisolone tablets for patients with IgA nephropathy is unsatisfactory, which requires combined treatment with other drugs to further enhance the therapeutic effect of patients.

Traditional Chinese medicine has unique advantages in the treatment of IgAN, which can effectively attenuate the symptoms of patients and delay the progress of IgAN. However, the current research on the intervention of traditional Chinese medicine on IgAN mainly focuses on antioxidative stress, inhibiting the proliferation of mesangial cells and mesangial hyperplasia, protecting the tubulointerstitium, and delaying renal interstitial fibrosis. Many studies have confirmed that the treatment of IgA nephropathy with integrated traditional Chinese and western medicine has achieved satisfactory results [6]. In traditional Chinese medicine, IgA nephropathy is classified as “kidney wind,” “consumptive disease,” “low back pain,” “edema,” “hematuria,” etc., and the most common clinical syndromes of the disease are spleen-kidney qi deficiency syndrome, qi-yin deficiency syndrome, liver-kidney yin deficiency syndrome, etc. Among them, spleen-kidney qi deficiency syndrome is the most common. The etiology and pathogenesis of the disease may be the invasion of wind-heat-dampness pathogens, injury from diet and fatigue, and internal heat due to yin deficiency in the body. The kidney is the innate root, the spleen is the acquired root, and spleen-kidney qi deficiency causes qi not to absorb blood or blood stasis. And, the disease is a kind of deficiency symptoms, the primary deficiency is the deficiency of the spleen and kidney, and the symptoms are damp heat and blood stasis. Therefore, the treatment of the disease is to nourish qi and nourish yin, strengthen the spleen and kidney, promote blood circulation, and remove blood stasis.

Huangkui capsule is a TCM preparation composed mainly of Flower of Sunset Abelmoschus, which has the effects of clearing heat, relieving dampness, reducing swelling, and expelling toxins. The results by Ambarsari et al. [7] encouraged the combination of Chinese and western drugs for IgA nephropathy. Accordingly, this study was undertaken to investigate the clinical efficacy of Huangkui capsule plus methylprednisolone for IgA nephropathy, as well as its effect on renal function and serum inflammatory factors.

## 2. Materials and Methods

### 2.1. Participants

A total of 80 patients with IgA nephropathy admitted to our hospital from April 2019 to December 2021 were recruited and assigned (1 : 1) to receive either conventional drugs + methylprednisolone tablets (observation group) or conventional drugs + methylprednisolone tablets + Huangkui capsules (experimental group), with 40 patients in each group. The randomization was carried out using an online web-based randomization tool (freely available at https://www.randomizer.org/). For concealment of allocation, the randomization procedure and assignment were managed by an independent research assistant who was not involved in the screening or evaluation of the participants. Prior to enrollment, the study obtained the informed consent of the patients, and the protocol was approved by the hospital ethics committee (SD-ER20190708). All procedures were performed in line with the ethical guidelines of the Declaration of Helsinki.

The original sample size calculation estimated that 40 patients in each group would be needed to detect a 3-point difference between groups in a 2-sided significance test with a power of 0.8 and an alpha error level of 0.05.

#### 2.1.1. Inclusion Criteria

Patients with a diagnosis of IgA nephropathy that was confirmed by clinical Chinese and western medicine-related test results and who provided written informed consent were included.

#### 2.1.2. Exclusion Criteria

(1) Those with active bleeding disease; (2) those who are allergic to the drugs used in this study; (3) breastfeeding and pregnant women; (4) secondary IgA nephropathy induced by lupus nephritis, purpuric nephritis, etc.; (5)) hypertensive nephropathy, crescentic nephritis, acute interstitial nephritis, and other acute renal insufficiencies; (6) patients with renal artery stenosis and serious organs dysfunction; (7) received glucocorticoid dose >20 mg/d for >4 weeks in the past 3 months; (8) patients who have received immunosuppressive and cytotoxic treatment for more than 4 weeks in past 3 months; (9) patients with malignant tumors or a history of malignant tumors; (10) severe gastrointestinal diseases; (11) acute central nervous system diseases.

### 2.2. Treatment Methods

Patients in both groups received 30 mg of dipyridamole tablets (National Drug Administration: H20066585) thrice daily, 90 mg of valsartan capsules (National Drug Administration: H20010811) daily, and 45 mg of methylprednisolone tablets (National Drug Administration: H2220245) daily as an initial dose and 8 mg daily as a maintenance dose. All the above-given drugs were administered orally, and the duration of treatment was 10 weeks.

Patients in the experimental group additionally received 2 g of Huangkui capsules (National Drug Administration: Z19990040) thrice daily. The duration of treatment was 10 weeks.

### 2.3. Outcome Measures

1. Clinical efficacy: cured: clinical symptoms completely disappeared, with 24 h urine protein amount of <0.3 g/d; effective: the patient's symptoms basically disappeared, and the 24 h urine protein amount decreased by more than 50% compared with the previous levels; ineffective: there were no improvement or even aggravation of clinical symptoms.
2. Renal function indices: 3 mL of morning fasting venous blood was collected from both groups and centrifuged to obtain the serum for the following assays. The levels of serum creatinine and serum urea nitrogen were determined by an automatic biochemical analyzer (model: 7677–920), the levels of fibrinogen were determined by latex-enhanced immunoturbidimetric method, and the levels of 24 h urine protein were determined by ophthalmic triphenyl red colorimetric method.
3. Serum inflammatory factors: serum inflammatory factors, including tumor necrosis factor-*α* (TNF-*α*), interleukin-6 (IL-6), and monocyte chemoattractant protein-1 (MCP-1) were measured by enzyme-linked immunosorbent assay.
4. The lgA level was detected by nephelometry using the automatic special protein analyzer of Beckman–Coulter Company. The expressions of MCP-1 and IL-6 were detected by ELISA.
5. Adverse events: adverse events during treatment were recorded, including electrolyte disturbances, abdominal discomfort, gastrointestinal bleeding, and infections.

### 2.4. Statistical Analysis

SPSS21.0 was used for data analyses. Normally distributed measurement data were expressed as ($\bar{x}$ ± *s*) and analyzed using the independent *t*-test, and count data were expressed as *n*(%) and analyzed by the chi-square test. *P* < 0.05 was used as a cut-off for statistical significance.

## 3. Results

### 3.1. Patient Characteristics

There were 24 males and 16 females in the observation group, aged 21–68 years, with a disease duration of 5–57 months. There were 27 males and 13 females in the experimental group, aged 25–71 years, with an illness duration of 3–55 months. The two groups' patient characteristics were equivalent (*P* > 0.05) (Table 1).

### 3.2. Clinical Efficacy

The experimental group showed a significantly higher clinical efficacy versus the observation group (*P* < 0.05) (Table 2).

### 3.3. Renal Function Indices

After therapy, patients in the experimental group had significantly lower blood creatinine, serum urea nitrogen, fibrinogen, and 24 h urine protein levels than those in the control group (*P* < 0.05) (Table 3).

### 3.4. Inflammatory Factor Levels

After treatment, the experimental group showed lower levels of TNF-*α*, IL-6, and MCP-1 than the observation group (*P* < 0.05) (Table 4).

### 3.5. Adverse Events

The disparities in adverse occurrences between the two groups did not meet the statistical threshold (*P* > 0.05) (Table 5).

## 4. Discussion

IgA nephropathy is a primary glomerular disease characterized by the deposition of IgA with other immunoglobulins in the glomerular thylakoid region [8]. The risk factors for the progression of the disease include glomerulosclerosis, serum creatinine, hypertension, hematuria, and proteinuria. Therefore, the main clinical manifestations of progressive (severe) IgA nephropathy are interstitial fibrosis, renal tubular atrophy, renal tissue light microscopy changes with glomerular sclerosis or glomerular sclerosis, persistent massive proteinuria, elevated serum creatinine, and primary glomerulonephritis with hypertensive lesions. Severe IgA nephropathy is Lee's grade III or higher, with significantly increased renal interstitial fibrosis, renal tubular damage, increased glomerular sclerosis or crescent formation and other lesions, renal biopsy pathology with necrosis, hypertension, and protein urine (>1.0 g/24 h) as the main clinical manifestation [9]. The current clinical treatment for patients with IgA nephropathy primarily involves pharmacological interventions for blood pressure management, dilation, decongestion, diuresis, and immunosuppression [10]. Therefore, angiotensin-converting enzyme inhibitors, angiotensin receptor inhibitors, lipid-lowering drugs, or glucocorticoids are commonly used for IgA nephropathy. Angiotensin II receptor blockers or angiotensin-converting enzyme inhibitors in the treatment of IgA nephropathy can effectively control urinary protein and improve renal function, but the effect is unsatisfactory [11].

Glucocorticoids are commonly used in the treatment of patients with IgA nephropathy as they improve the permeability of the glomerular membrane, facilitate urination, and reduce the level of urinary protein. Stangou et al. [12] found that glucocorticoids also inhibited the inflammation and immune response in patients with IgA nephropathy. Methylprednisolone is a medium-acting glucocorticoid that regulates the cellular and humoral immunity, thereby enhancing the immune function of the patients [13]. It has been suggested that methylprednisolone markedly increased the isotonicity of the patient and exhibited anti-immune, anti-inflammatory, and antitoxic effects with mild side effects of water and sodium retention [14]. Methylprednisolone is a medium-acting glucocorticoid with a moderate biological half-life, which prevents drug accumulation [15]. However, the reduced renal function of patients with IgA nephropathy causes an aggressive decline of the body's functional system, and glucocorticoids plus conventional drugs fail to manage urinary protein and increase the risk of disease progression. Wang et al. also indicated that the effectiveness of conventional drugs plus methylprednisolone tablets for IgA nephropathy is suboptimal and requires additional treatment in combination to improve therapeutic outcomes.

Studies have shown that the method of nourishing qi and tonifying the kidney can significantly reduce the fusion of podocytes, reduce the deposition of IgA in the mesangium of the glomerulus, improve the proliferation of the mesangial matrix, and delay the glomerular sclerosis [16]. Damp heat affects the entire pathological process of IgA nephropathy and is significantly correlated with the degree of glomerular disease, the degree of mesangial proliferation, the degree of immune complex deposition, and the degree of tubulointerstitial damage [17]. Pathological changes such as increased extracellular matrix accumulation, vascular loop occlusion, focal or segmental glomerulosclerosis, and interstitial fibrosis are in line with the characteristics of TCM [18]. During the pathogenesis of IgA nephropathy, damp heat, and blood stasis are not only important pathological products but also secondary causes of development and outcome [19]. Therefore, clearing heat and removing dampness, promoting blood circulation and removing blood stasis, and eliminating symptoms should be used throughout the treatment of IgAN. Huangkui capsules are commonly used in TCM as an adjunct for renal diseases and are characterized as sweet in taste, cold in nature, and nontoxic [20]. The main component of Huangkui capsule is flower of sunset abelmoschus, containing active ingredients such as myricetin, quercetin-3-robinobioside, quercetin-3-glucoside, quercetin, and hyperin. Lv et al. [21] found that Huangkui capsules effectively inhibit the glomerular immunity of the body, reduce the inflammatory response, suppress platelet aggregation, and alleviate kidney function impairment. Besides, Huangkui capsule also scavenges oxygen free radicals and lowers urinary protein, blood urea nitrogen, and libido [22].

The results of this study showed that the experimental group had a significantly higher efficiency and higher levels of serum creatinine, serum urea nitrogen, fibrinogen, and 24-h urine protein than the observation group, indicating that Huangkui capsule plus methylprednisolone is clinically effective for IgA nephropathy and improves the renal function of the patient. The reason may be that the anti-inflammatory effect of methylprednisolone mitigates renal injury [23], and the effective removal of oxygen free radicals and reduction of urinary protein by Huangkui capsules strengthen the attenuation of renal injury. TNF-*α* and IL-6 are both common proinflammatory factors in IgA nephropathy. MCP-1, a low molecular weight chemokine and a soluble basic protein, is expressed in glomerular endothelial cells, immune cells, renal tubular epithelial cells, renal mesangial cells, renal interstitial fibroblasts, and other normal kidneys cells in after being stimulated. Studies have shown that the overexpression of MCP-1 can also cause damage to renal tissue and play an important role in the progression of IgA nephropathy. MCP-1 can promote the synthesis and secretion of ICAM-1 by activating the protease-1 pathway, thereby participating in the inflammatory response. MCP-1 can stimulate related cells to promote the release of superoxide anions and lysosomal enzymes, resulting in direct damage to the kidney; it can also activate and promote monocytes to secrete TGF-*β* and other factors that can cause cell fibrosis, thereby promoting glomerular sclerosis, causing fibrosis of the renal interstitium, and ultimately the progression of IgA nephropathy to end-stage renal failure [24, 25]. So these three inflammatory factors are often tested clinically to reflect the magnitude of inflammatory damage in the patient [26]. The results of this study showed that the serum TNF-*α*, IL-6 and MCP-1 levels in the experimental group were significantly lower than those in the observation group after treatment, which indicated that Huangkui capsules plus methylprednisolone effectively reduced the inflammatory response in patients with IgA nephropathy. The reason may be that flavonoids such as myricetin and quercetin are antibacterial and anti-inflammatory improve the immunity of patients and alleviate the inflammatory response of patients [27]. Considering the above-given theory, it is preliminarily speculated that the mechanism of action of Huangkui capsules may realize via inhibiting the expression of MCP-1 and inflammatory factors MCP-1 and IL-6, thereby reducing urinary protein excretion and exerting a certain renal protective effect.

In addition, the two groups showed no significant differences in terms of adverse events, suggesting a manageable safety of Huangkui capsules plus methylprednisolone in the treatment of patients with IgA nephropathy. However, this safety outcome may also be attributed to the limitations of the present study, including short followup and the small number of samples. Future multicenter trials with a larger sample size and long-term followup will be conducted to provide more reliable data [28, 29].

Although it has certain guiding significance, there are still several limitations: the number of samples is small, the observation period is short, and there is no long-term followup. Therefore, the strategy should be improved by expanding larger samples, so as to provide more clinical evidence for the research and application of this method.

## 5. Conclusion

Huangkui capsule plus methylprednisolone offers a viable treatment alternative for IgA nephropathy by significantly improving the renal function of patients, effectively reducing the inflammatory response, and providing a high safety profile, which shows great potential for clinical application.

## Figures and Tables

**Table 1:** Patient characteristics [$\bar{x}$ ± *s*, *n*(%)].

	Observation (*n* = 40)	Experimental (*n* = 40)	*t*/*x*^2^	*P*-value
Gender			0.487	0.485
Male	24	27		
Female	16	13		
Age (year)	21–68	25–71		
Mean age (year)	42.84 ± 10.35	43.24 ± 10.59	−0.171	0.865
Disease course (month)	5–57	3–55		
Mean disease course (month)	23.48 ± 11.59	22.94 ± 11.46	0.21	0.834

**Table 2:** Clinical efficacy [*n*(%)].

	Observation (*n* = 40)	Experimental (*n* = 40)	*x* ^2^	*P*
Cured	14	27		
Effective	16	12		
Ineffective	10	1		
Total efficacy (%)	30 (75%)	39 (98%)	8.538	0.003

**Table 3:** Renal function indices ($\bar{x}$ ± *s*).

Group	*N*	Serum creatinine (*μ*mol/L)		Serum urea nitrogen (mmol/L)		Fibrinogen (g/L)		24 h urine protein (g)	
		Before treatment	After treatment	Before treatment	After treatment	Before treatment	After treatment	Before treatment	After treatment
Observation	40	117.65 ± 16.38	95.84 ± 10.26	6.34 ± 2.35	5.47 ± 1.81	5.16 ± 1.27	4.19 ± 0.81	6.51 ± 2.97	3.75 ± 0.92
Experimental	40	117.59 ± 16.45	72.51 ± 8.19	6.33 ± 2.32	4.57 ± 1.26	5.18 ± 1.25	3.25 ± 0.54	6.48 ± 2.99	1.63 ± 0.54
*t*-value	—	0.016	11.24	0.019	2.581	−0.071	6.107	0.045	12.569
*P*-value	—	0.987	<0.001	0.985	0.012	0.944	<0.001	0.964	<0.001

**Table 4:** Inflammatory factor levels ($\bar{x}$ ± *s*).

Indices	Timepoint	Observation (*n* = 40)	Experimental (*n* = 40)	*t*-value	*P*-value
TNF-*α* (ng/L)	Before treatment	41.57 ± 7.39	41.62 ± 7.43	−0.03	0.976
	After treatment	37.74 ± 7.68	15.69 ± 6.21	14.12	<0.001

IL-6 (ng/L)	Before treatment	38.82 ± 7.11	38.68 ± 7.19	0.088	0.93
	After treatment	36.31 ± 5.76	17.68 ± 5.88	14.315	<0.001

MCP-1 (ng/L)	Before treatment	152.78 ± 16.84	151.67 ± 16.49	0.298	0.766
	After treatment	126.24 ± 15.33	102.09 ± 15.48	7.011	<0.001

**Table 5:** Adverse events [*n*(%)].

	Observation (*n* = 40)	Experimental (*n* = 40)	*x* ^2^	*P*-value
Electrolyte disorders	0	1	—	—
Abdominal discomfort	1	1	—	—
Gastrointestinal bleeding	0	0	—	—
Infections	1	1	—	—
Total incidence (%)	2 (5%)	3 (8%)	0.213	0.644

## Data Availability

The datasets used and analyzed during the current study are available from the author upon reasonable request.
